# Preparation and Spectroscopic, Thermal, and Mechanical Characterization of Biocomposites of Poly(butylene succinate) and Onion Peels or Durum Wheat Bran

**DOI:** 10.3390/ma16206799

**Published:** 2023-10-21

**Authors:** Emil Sasimowski, Marta Grochowicz, Łukasz Szajnecki

**Affiliations:** 1 Department of Technology and Polymer Processing, Faculty of Mechanical Engineering, Lublin University of Technology, 20-618 Lublin, Poland; e.sasimowski@pollub.pl; 2 Department of Polymer Chemistry, Institute of Chemical Sciences, Faculty of Chemistry, Maria Curie-Sklodowska University, 20-614 Lublin, Poland; lukasz.szajnecki@mail.umcs.pl

**Keywords:** agro-flour filler, agro-waste materials, biofiller, composite, injection molding, mechanical properties, PBS, thermal properties, thermo-mechanical properties

## Abstract

The utilization of plant based fillers: onion peels (OP) and durum wheat bran (WB) to obtain sustainable biocomposite materials with poly(butylene succinate) (PBS) is presented in this paper. The biocomposites were first obtained in pellet form by extrusion method and then injection moldings were made from the pellets. Two kinds of biocomposites were fabricated containing 15% and 30% wt. of OP or WB. Additionally, pure PBS moldings were prepared for comparative purposes. The effect of the filler type and its amount on the chemical structure, density, thermal, and thermo-mechanical properties of the fabricated composite samples was studied. Fourier-transform infrared spectroscopy results showed that the composite preparation method had no effect on the chemical structure of composite components, but weak interactions such as hydrogen bonding between OP or WB and PBS was observed. The addition of OP or WB to the composite with PBS reduced its thermal stability in comparison with pure PBS, all studied composites start to degrade below 290 °C. Additionally, the mechanical properties of the composites are worse than PBS, as the impact strength dropped by about 70%. The deterioration of tensile strength was in the range 20–47%, and the elongation at maximum load of the composites was in the range 9.22–3.42%, whereas for pure PBS it was 16.75%. On the other hand, the crystallinity degree increased from 63% for pure PBS to 79% for composite with 30% wt. of WB. The Young’s modulus increased to 160% for composition with 30% wt. of OP. Additionally, the hardness of the composites was slightly higher than PBS and was in the range 38.2–48.7 MPa. Despite the reduction in thermal stability and some mechanical properties, the studied composites show promise for everyday object production.

## 1. Introduction

Due to growing public awareness of the environmental impact of traditional plastics, the biodegradability of polymer plastics is now of increasing importance [1,2]. Replacing petroleum-based plastics with biodegradable polymers that meet the requirements for a given products is a key approach to decrease the amount of non-degradable plastics in the environment [3,4]. Biodegradable plastics include, polylactide (PLA), poly(hydroxyalkanoates) (PHAs), poly(butylene adipate terephthalate) (PBAT), thermoplastic starch (TPS), and poly(butylene succinate) (PBS). Two sources of biodegradable polymers can be distinguished: natural and synthetic. Polymers of natural origin are directly obtained from plants, animals, or microorganisms. Synthetic biodegradable polymers can be synthesized from petroleum-based monomers or from bio-based monomers [5,6,7]. One of the more promising biodegradable plastic is PBS. Its good thermal and mechanical properties, lack of cytotoxicity in combination with biodegradability makes it suitable for use in many applications [5]. PBS is usually produced by polycondensation of succinic acid (SA) and 1,4-butanediol (BDO). Monomers for polycondensation may be of petroleum origin or produced as a result of bacterial fermentation [6]. Unfortunately, its manufacturing costs are much higher compared to traditional petrochemical plastics [7,8].

The main way to reduce the price of biodegradable polymer materials as well as to modify their properties is to use natural fillers such as wood chips [9], ground bran of wheat cereals [10,11,12], rice [13], ground coffee beans [14,15,16], shells of various nuts [17,18,19], natural fibers [20] (cellulose, hemp, flax, sugar cane, bamboo) and many other types of agricultural waste [21,22,23,24]. The latest works published in 2023 year on PBS compositions concerns the use of fillers such as biochar [25,26,27], flax and pineapple leaf fiber [28,29], graphene nanoplatelets and starch-based materials [30], biogenic wine by-products [31], plant fibers (jute, kenaf, flax, and hemp) [32], rice straw fiber [33], and nanofibrillated cellulose [34].

Natural fillers are waste products generated mainly by agriculture and the food industry. They are widely available lignocellulosic materials and there are often problems with their storage and disposal. Considering the injection methods of producing composites, lignocellulosic fillers (LCF) have one major disadvantage. Their thermal stability is low, often degrading at about 130–150 °C. This affects the temperature range for extrusion and injection processes and reduces the thermal resistance of the composites obtained with them. Thus, the matrix polymer should have a melting point lower than the decomposition temperature of the filler. Due to its melting point of about 115 °C [12], PBS is an appropriate polymer matrix material for LCF. Moreover, the mechanical strength of biocomposites with LCF significantly decreases with the filler content [35,36,37], which results from the strength of interactions at the polymer matrix/LCF interface. Significant differences in the hydrophobicity/hydrophilicity of the matrix and filler reduce the possibility of chemical interactions between the composite components. However, PBS has a hydrophilic character (water wetting angle of 70°) [38], and this is one of the reasons why its interaction with LCF is so good, as compositions with a very high filler content have an acceptable mechanical strength [11].

Hence, considering the need to look for new solutions in the synthesis of biodegradable polymer composites that are cheaper than pure biodegradable polymers, but retain the required set of mechanical and thermal properties, it was decided to obtain PBS compositions with onion peels (OP) and wheat bran (WB). To the best of our knowledge, there are no studies in the available literature regarding the composition of PBS with the addition of ground (OP). According to Food and Agriculture Organization of the United Nations, in 2021 the global production of onion was about 107 million tones [39]. Onion ranked as the second most produced vegetable—after tomatoes. OP is waste material from onion processing and due to the high production of onion it can be considered as a potential agro-waste filler for composite synthesis. As presented in the study of Rhim and co-workers, OP is built mainly from α-cellulose (41.1%), lignin (38.9%), hemicelluloses (16.2%), and extractives (3.8%), among which phenolic compounds, flavonoids and flavones are present [40]. The second biofiller used in this study was WB derived from durum wheat (*Triricum durum*). Previous studies by the authors addressed PBS with common WB and not durum wheat bran, which was used in this case [11,12]. WB is the waste material in flour production. It consists mainly of lignocellulosic materials including cellulose, lignin, hemicellulose, and also phytic acid, oligosaccharides, non-starch polysaccharides, as well as fats and proteins [41,42,43]. The main goal of this paper was to investigate the relationship between the properties of polymer compositions based on PBS and the content and type of fillers of plant origin, OP and WB. The conducted studies include the determination of chemical structure, density, hardness, thermal properties, impact strength, tensile strength, Young’s modulus, and elongation at the break of the investigated biodegradable PBS compositions.

## 2. Experimental

### 2.1. Production of Biocomposition and Measurement Samples

**Figure 1:**
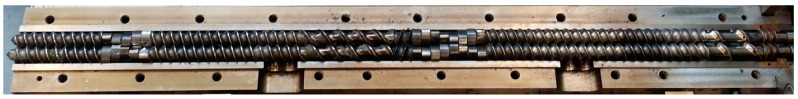
View of an open plasticizing system of the extruder with the processing screws.

The manufacturing process of polymer compositions was carried out by extrusion with granulation. The tested plant-based fillers, WB and OP, were dried in a laboratory dryer at 60 °C for 24 h. The dry fillers and PBS pellets were premixed with the use of a frame mixer after weighing. After that, the obtained mixture was processed using the EHP-2 × 20 Sline co-rotating twin-screw extruder (Zamak Mercator, Skawina, Poland). The machine uses segmented processing screws with a diameter of 20 mm and a working part-to-diameter ratio of 40. In Figure 1, the image of the screw configuration used in the study is presented. It contains two kneading zones, the first consisting of eight double-lobe cams (4D length), and the second of five double-lobe cams (2.5D length). In both kneading zones, the cam angle was 30° and the direction of inclination of the kneading elements was clockwise. A barrier element was located after the first of the kneading zones, with the opposite winding direction, behind which there was a zone of gravity degassing. The extrusion process was conducted using an extrusion head with a nozzle with two 3 mm diameter round holes. The composite extrusion process was carried out at the extruder’s gravity feed and screw speed of 125 min^−1^. The plasticizing system had nine zones and the temperature in all of them was set to 145 °C. The extrusion head had a temperature of 140 °C. The obtained extrudate strands were cooled in a water bath and cut using a granulator, respectively. The produced pellets were then dried in a laboratory dryer at 60 °C for 24 h prior to further processing. The measured moisture content of the composition pellets after drying was below 1.5%.

Injection moldings of the prepared composition pellets were manufactured using the injection method. Dog bone specimens were made with the use of a screw injection molding machine (Arburg Allrounder 320C, Lossburg, Germany), which has a dual cavity mold. The obtained sample dimensions were in accordance with ISO 294-1:-07 77 [44]. Taking into account the possibility of thermal decomposition of the natural components of the biocomposition, lower processing temperatures than those recommended for PBS alone were used. The injection conditions of the measurement samples are shown in Table 1.

### 2.2. Materials

PBS for injection molding, with the trade name BioPBS FZ71 PB [45] (PTT MCC BIOCHEM Co., Ltd., Bangkok, Thailand) in the form of pellets was used as a matrix material. It should be emphasized that PBS was produced from bio-based succinic acid and butane-1,4-diol [5,46,47]. The WB from durum wheat (*Triricum durum*) that was used as a filler came from PZZ Lubella GMW (Lublin, Poland). The WB arrived in flake form and was up to 0.5 mm in size. The OP filler, in the form of thin yellow-brown peels, came from a local fruit and vegetable processing plant. The delivered peels ranged in size from a few to a few dozen millimeters. For this reason, they were ground with a knife mill before being introduced into the biocomposition.

### 2.3. Research Program and Methodology

Experiments were performed according to the adopted research plan, using 3 different mass fractions of 0%, 15% and 30% wt. filler in the biocomposition and 2 different types of natural fillers: OP or WB. The assays adopted in this study for the measurement samples obtained by injection molding from previously manufactured biocomposition pellets and from pure PBS pellets are shown in Table 2.

The particle morphology of OP and WB measurements were determined using an automatic optical Morphologi G3 particle size and particle shape image analyzer (Malvern) equipped with a dispersion unit (SDU) of the powdered sample of the test substance. Before the measurements, 19 mm^3^ of the filler sample was sprayed with an SDU (spray pressure = 1 bar, spray time = 40 ms, particle fall time = 120 s) over an area of 60.79 cm^2^. The analysis of particles present in 4 areas (A, B, C and D), each measuring 20 mm × 20 mm, was then performed.

The following parameters were determined to describe the morphology of the particles: visual projected area (*P_A_*) of the particle, circle equivalent diameter (*CE_diam_*), aspect ratio (*AR*), and circularity (C) [48].

*CE_diam_* is defined as the diameter of a circle with the same area (*C_A_*) as the projected area of the particle image (*P_A_*). Very differently shaped particles may be characterized as identical by using *CE_diam_* only because they have similar projected 2D areas. Thus, information about the shape of the particles is required.

The aspect ratio was calculated according Equation (1) as a ratio of the particle width (*P_W_*) to the particle length (*P_L_*):

(1) $$AR=\frac{P_{W}}{P_{L}}$$

The *AR* values range from 0 to 1. The more elongated the shape of the particle, the smaller its *AR*.

Circularity (*C*) was calculated from Equation (2) as the ratio of the perimeter of a circle with the same area (*C_P_*) as the particle divided by the perimeter of the particle image (*P_P_*).

(2) $$C=\frac{C_{P}}{P_{P}}=\frac{2\sqrt{\pi \times P_{A}}}{P_{P}}$$

**Figure 2:**
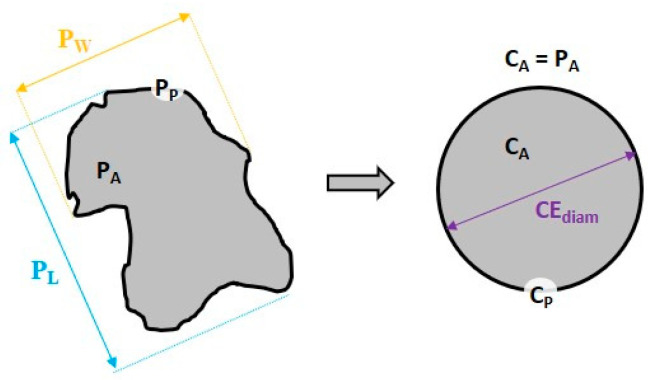
A 2D sketch of the 3D particle and the circle equivalent idea.

Circularity is in the range 0–1. Circularity is sensitive to both elongation and surface roughness. Figure 2 presents the idea of circularity calculation.

The standard density *ρ* [g/cm^3^] of biocomposites was assessed according to ISO 1183-1 A [49]. The immersion method with water as an immersion medium was used.

The TENSOR 27 FTIR spectrophotometer (Bruker, Billerica, MA, USA) with an attenuated total reflectance (ATR) attachment containing a diamond crystal was used for FTIR analysis of the samples. Each spectrum was taken in the range 600–4000 cm^−1^ with a resolution of 4 cm^−1^. A total of 32 scans were made per spectrum.

A differential scanning calorimetry (DSC) analysis was performed using the 204 F1 Phoenix DSC scanning calorimeter (NETZSCH, Günzbung, Germany) and NETZSCH Proteus 6 data processing software (NETZSCH, Günzbung, Germany), in compliance with the ISO 11357-1:2023 standard [50]. The DSC experiment was conducted for three cycles: heating (I) from −100 °C to 140 °C, cooling from 140 °C to −100 °C, and heating (II) from −100 °C to 140 °C (the heating and cooling rate was set to 10 °C/min). The samples had a mass of about 10 mg. Aluminum crucibles with a pierced lid were used. The obtained DSC results were used to determine the degree of crystallinity *X_c_*, melting enthalpy Δ*H_m_*, melting point *T_m_*, crystallization temperature *T_c_*, and glass transition temperature *T_g_* (the curve inflection point in the glass transition region) for the fabricated composite materials. The degree of crystallinity was calculated according to Equation (3):

(3) $$X_{c}=(\frac{∆H}{\left(1-u\right)\times {∆H}_{100\%}})\times 100\%$$

assuming that for PBS, Δ*H*_100%_ = 110.3 J/g [51].

The STA 449 F1 Jupiter thermal analyzer (NETZSCH, Günzbung, Germany) was used to perform a thermogravimetric analysis (TG). The measurements were made for the temperature range 40–700 °C, in a synthetic air atmosphere. The gas flow rate was 25 mL/min. The sample mass was approx. 12 mg. Open Al_2_O_3_ crucibles were used. From the TG curves, characteristic temperatures of 5 and 50% of mass loss (*T*_5%_, *T*_50%_) were determined, whereas the temperatures of the maximum rate of mass loss (*T_max_*_1_, *T_max_*_2_, *T_max_*_3_) were derived based on DTG curves.

The ball indentation method was used to measure hardness of the biocomposite samples. The hardness measurements were made using the HPK 8411 hardness tester with a ball-shaped indenter of 5 ± 0.025 mm in diameter. The measurements were performed in compliance with the ISO 2039-1:2001 standard [52].

A type 639F impact hammer from Cometech Testing Machines (Taizhong, Taiwan) was used to perform notched Charpy impact tests according to the ISO 179-2:2020 standard [53]. A B-type notch was made in the surface of the samples. The specimens were tested in a position corresponding to the edge impact of a hammer, whose maximum energy was 5.093 J.

The strength properties of the samples were tested in accordance with ISO-2 [54] at a tensile speed of 50 mm/min using the Z010 Zwick Roell testing machine (Ulm, Germany). Young’s modulus E [MPa], tensile strength *σ* [MPa]. and elongation at maximum load *ε_m_* [%] were calculated.

The obtained results were compared by analysis of variance (ANOVA) to verify whether they met the assumptions of this analysis. This was followed by a Tukey’s post-hoc test. The significance level of *p* = 0.05 was assumed in the applied analyses. The graphs present average values and standard deviations of the obtained test results.

## 3. Results

### 3.1. Physical and Structural Properties

#### 3.1.1. Morphology of the Natural Fillers

**Figure 3:**
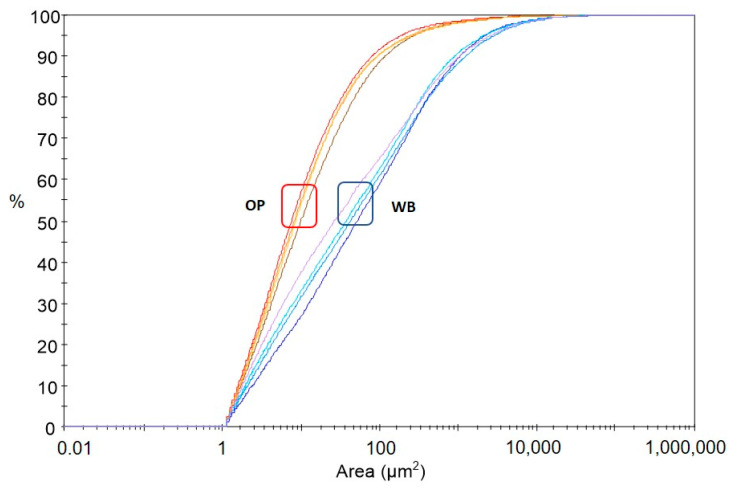
Frequency and oversize curves for different particle sizes. Red lines—OP; blue lines—WB.

**Figure 4:**
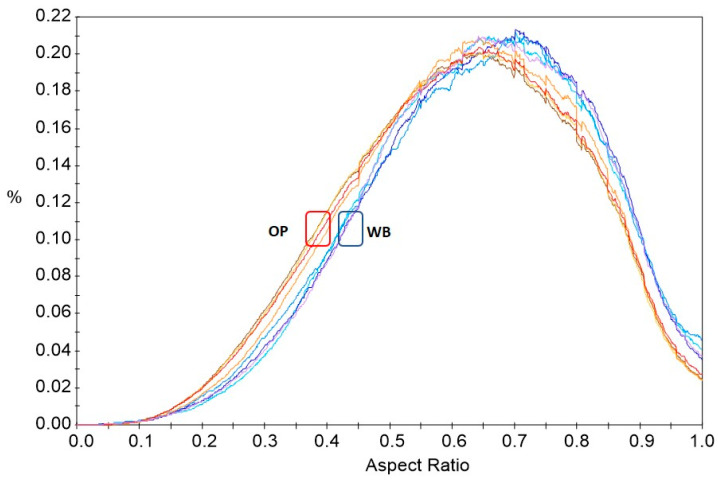
Distribution of the aspect ratio (AR) parameter for samples of OP (red lines) and WB (blue lines).

A morphological analysis, i.e., determining the size, shape, and number of particles present in the sample under study, can be a remarkably important yet difficult issue—especially if the particles are not spherical and have an irregular shape, which is exactly the case with the particles of onion peel and wheat bran used to fabricate the composition for this study [55]. One way to describe the morphology of particles in a sample is through microscopic image analysis. The point of the measurement is to replace the three-dimensional shape of the particle (3D) with its two-dimensional (2D) projection on the plane. The recorded image is subject to analysis, and the results make it possible to calculate the parameters used to describe the size and shape of the particles [56]. To analyze the morphological features of OP and WB, parameters such as area, aspect ratio, and circularity were selected and calculated from the images presented in Supplementary Figures S1 and S2. From the cumulative particle size distribution curves of ground OP and WB samples, it is apparent that they have a similar pattern for different measurements of the same filler (Figure 3). In contrast, the particle size distribution of WB samples differs markedly from the corresponding distribution for OP samples. The mean area of WB is higher than for OP (Table 1, SI). The course of the AR parameter distribution curves for OP is also noticeably different from that for WB curves (Figure 4). This proves the existence of differences in particle shape in OP and WB samples.

#### 3.1.2. Density

**Figure 5:**
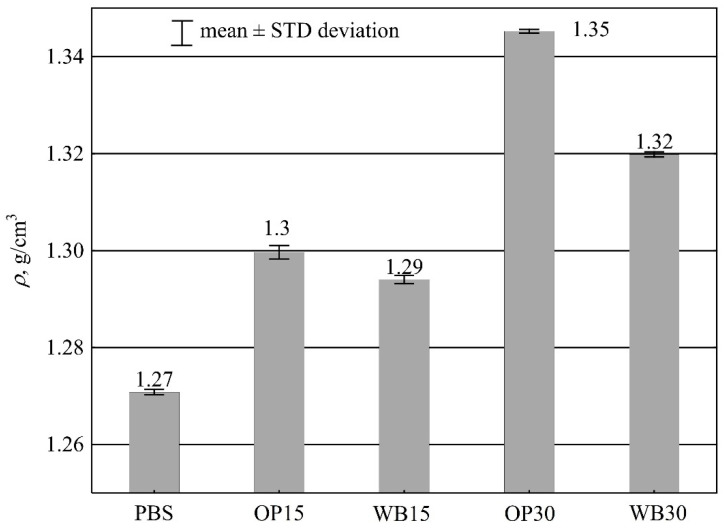
The density *ρ* of PBS and its composites with OP or WB.

As shown below in Figure 5, the density of obtained moldings is strongly affected by the both type and mass content of the fillers introduced into the composition. Significantly higher density values were observed when OP was used instead of WB. The density of the samples made with pure PBS was the lowest, averaging *ρ* = 1.271 g/cm^3^. The addition of 15% and 30% wt. of WB with a density of 1.450 g/cm^3^ increased the density of the polymer moldings by 0.023 g/cm^3^ (1.83%) and 0.049 g/cm^3^ (3.86%), respectively.

On the other hand, the use of OP in the same contents, whose density is 1.346 g/cm^3^, increases the density of the fabricated polymer composition moldings by 0.029 g/cm^3^ (2.27%) and 0.074 g/cm^3^ (5.86%), respectively.

Considering only the density values of the two fillers, the trend of changes in the density of the compositions should be the opposite, i.e., moldings containing WB should have a higher density than those containing OP. The likely reason for the observed differences is the greater ability of WB to absorb water compared with OP. This can also relate to the differences in the number of pores that formed in the moldings during the processing, due to the water evaporation contained in WB and OP.

The observed differences in density are quantitatively small, but statistically significant, which was confirmed by the ANOVA analysis and post-hoc test.

#### 3.1.3. Chemical Structure

**Figure 6:**
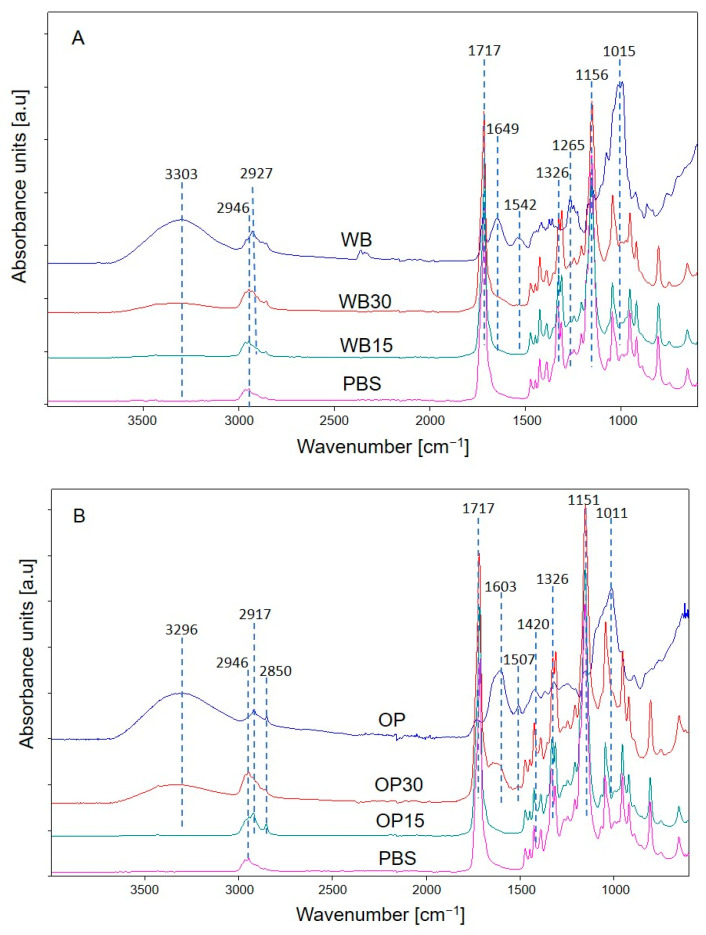
ATR-FTIR spectra of: (**A**) PBS, WB, and their composites; (**B**) PBS, OP, and their composites.

To confirm the chemical structure of molded PBS and its biocomposites containing WB and OP, an FTIR analysis was conducted. On the spectrum of neat PBS (Figure 6A,B), which belongs to polyesters, the following absorption band characteristics of ester bond vibrations are observed: at 1713 cm^−1^ (C=O); at 1263 cm^−1^, 1156 cm^−1^ and 1046 cm^−1^ (-C-O-C and -O-(C=O)). Additionally, the absorption bands at 2950–2850 cm^−1^ and at 1472 cm^−1^ and 1326 cm^−1^ derive from the vibration of the methylene groups. The absorption bands characteristic of polysaccharides are present on the bran spectrum (Figure 6A: the wide bands at 3303 cm^−1^ and at 1649 cm^−1^ come from the vibration of -OH groups; those in the range 2950–2850 cm^−1^ come from methyl and methylene groups vibration; 1716 cm^−1^—C=O groups vibration present in the carbonyl compounds contained in pectin and hemicellulose; at 1156 cm^−1^, 1015 cm^−1^ and 993 cm^−1^ they result from the vibration of C-O-C and C-O- groups [11,12]. In the spectra of WB15 and WB30 composites, absorption bands from both PBS and bran are visible. The intensity of bands originating from the organic filler increases with its content in the composite, and this is particularly evident for bands derived from hydroxyl groups vibration. One can also observe a slight shift in the position of carbonyl bands (to 1717 cm^−1^) compared to the PBS spectrum (1713 cm^−1^). This may suggest the existence of intermolecular interactions typical of weak hydrogen bonds between the carbonyl groups in the polymer chains and the hydroxyl groups in the bran polysaccharides [57,58]. The onion peel spectrum (Figure 6B) demonstrates that the absorption bands are derived from the vibrations of the following: hydroxyl groups (3296 cm^−1^ and a broadened band at 1603 cm^−1^), -C-O-C- groups (1011 cm^−1^) and the aromatic backbone of polyphenols (1507 cm^−1^, 1420 cm^−1^) [59]. As is visible in the spectra of OP15 and OP30, absorption bands from both polymer and filler components are present. Similarly to the WB composites, a shift in the position of the band of carbonyl groups to 1717 cm^−1^ is observed on the spectra of OP composites. For this case, it was possible to form weak hydrogen bonds between the carbonyl groups of PBS and the -OH groups of phenols contained in onion peels. In both the spectra of compositions with OP and WB, no additional absorption bands are visible compared to the spectra of fillers and PBS. Based on this, one can conclude that during the injection molding process neither a chemical reaction occurred between the components of the composition, nor did their possible thermal decomposition.

### 3.2. Thermal and Thermomechanical Properties

#### 3.2.1. DSC

**Figure 7:**
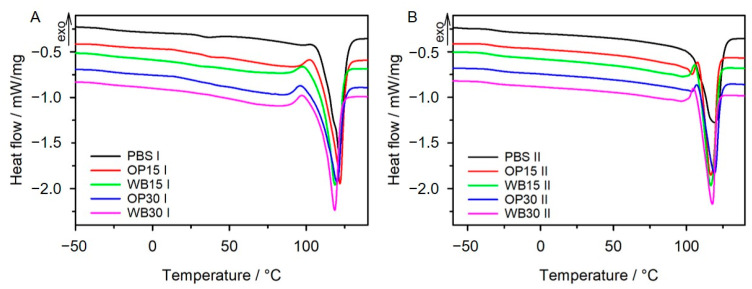
DSC thermograms (**A**) for the first (I) and (**B**) the second (II) heating scan of neat PBS and biocomposites with WB or OP.

In order to assess the thermal parameters and crystallinity degrees of PBS and biocompositions, DSC measurements were conducted in an inert gas atmosphere. To remove the thermal history of samples, analyses were performed in two heating cycles. In Figure 7, DSC thermograms are presented, whereas thermal parameters of the studied samples are given in Table 3. After eliminating the thermal history of the samples, their glass transition temperatures determined from the Heating II ranged from −33 °C to −29 °C. The *T_g_* value of neat PBS is higher than that of the compositions with 15% filler, regardless of the type of filler, while increasing the mass proportion of plant filler led to a small increase in *T_g_* compared to pure PBS. The introduction of 15% filler resulted in the separation of polymer chains and thus the reduction of *T_g_*. On the other hand, the higher *T_g_* at 30% filler content suggests that the increased amount of filler particles hindered secondary relaxation processes, also due to the formation of intermolecular interactions with the polymer, as indicated by the FTIR studies. The melting temperatures obtained in Heating I are higher than those in Heating II, but it can be seen from the thermograms that the melting peaks from the heating II cycle are narrower, indicating that the crystal structure is more ordered after the samples are melted. The Δ*H_m_* values calculated from the results of the heating I cycle are greater than those of the heating II. However, it should be kept in mind that both wheat bran and onion peels are materials capable of absorbing water, as revealed by the TG research. Consequently, the melting peak area from the heating I cycle is inflated by the effect of evaporation of the absorbed water from the samples. Besides, the DSC curves of WB and OP composites show broad endothermic peaks in the 50–100 °C range resulting from evaporation of surface-absorbed water by the samples. These peaks are not observed on the thermograms of the heating II cycle, which confirms that water evaporation has occurred. The *T_m_* of neat PBS is 118.5 °C. For composites with 15% fillers it drops slightly to 116.7 °C, most likely due to the presence of finer crystallites compared to pure PBS. At 30% filler content, *T_m_* values are slightly higher, suggesting that more homogeneous crystallites were formed during crystallization. In addition, the thermograms obtained for the composite materials in Heating II also reveal the presence of small endothermic peaks preceding the main melting peaks. They are clearer for the OP samples. Their presence is connected with the melting of smaller, less perfect crystallites. The degree of crystallinity for the composite samples is higher than that for the neat PBS sample and it increases with filler content, regardless of the filler type. This is different from the early result obtained for the composition of PBS with wheat bran [11,12]. In our earlier studies, *X_c_* decreased with the introduction of WB. The reason for this different behavior may be due to the type of PBS used to make the composition. In the current work, PBS with MFR of 22 g/10 min was used, while in previous work, MFR was 5 g/10 min. The *X_c_* values calculated for compositions with onion peels are about 10% smaller than those with wheat bran. The reason for this may be, firstly, due to the size of the filler particles—WB possesses a higher mean area than OP, so the contact of the polymer with the WB surface is greater and more crystallization nuclei are formed. Secondly, since in the structure of OP aromatic phenolic compounds are present, their surface is more hydrophobic than WB, which may result in less organization of the polymer chains and therefore a lower degree of crystallinity. The presence of an additional melting peak in the OP15 II and OP30 II thermograms confirms this supposition.

#### 3.2.2. Thermal Resistance

**Figure 8:**
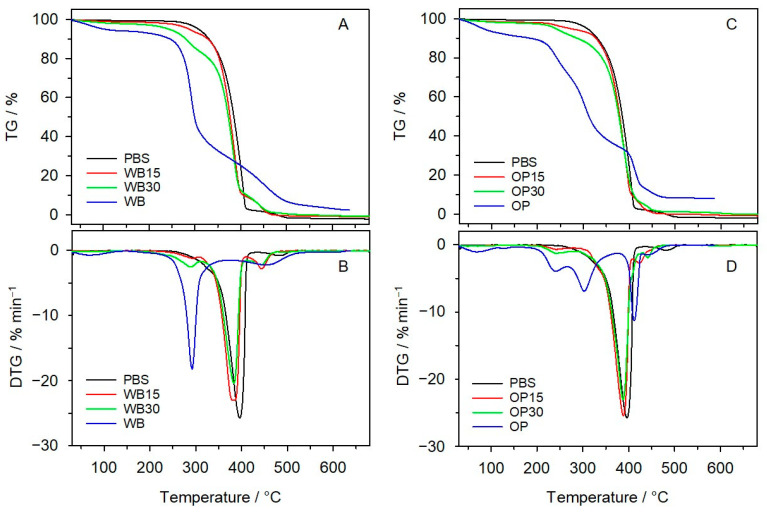
TG curves of PBS and biocompositions with WB (**A**) and OP (**C**) and the corresponding DTG curves of PBS and its biocomposites with WB (**B**) and OP (**D**).

**Figure 9:**
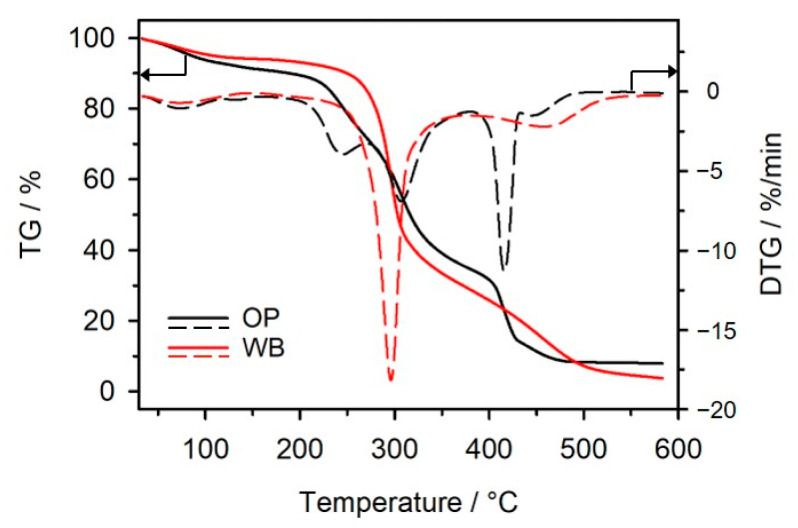
TG (solid lines) and DTG (dash lines) curves of WB and OP.

The thermal resistance and thermal decomposition route of the studied materials was assessed in oxidative atmosphere by means of thermogravimetric analysis. Table 4 shows the parameters characterizing their thermal behavior. An analysis of the TG curves in Figure 8 reveals that *T*_5%_, which was adopted as the temperature of the onset of decomposition, is the highest for PBS, while the composites with OP and WB show lower thermal resistance, which is below 300 °C. It decreases with an increase in the amount of vegetable filler and is lower by 20 °C for OP15 and by 10 °C for OP30 compared to the corresponding composites with WB. The reason for this observation is the lower thermal stability of OP than WB (Figure 9). The first weight loss visible on TG curves of OP and WB, starting from the lowest temperature to 110 °C, involves the evaporation of water absorbed in their structure—8.24% and 5.80%, respectively. *T*_5%_, determined for OP and WB after ignoring the weight loss associated with water evaporation, is 220 °C and 240 °C, respectively. WB undergoes thermal decomposition in two main stages with maximum rates at 296 °C and 459 °C, while OP undergoes thermal decomposition in three stages with *T_max_*_1_ at 245 °C, *T_max_*_2_ at 307 °C, and *T_max_*_3_ at 416 °C. Neat PBS shows two-stage decomposition with maximum rates at 396 °C and 480 °C. The DTG curves of the composites show three peaks, each overlapping with the DTG peaks of the individual components. The first weight loss (Δ *m*_1_) in the TG curves of the biocomposites is connected with the decomposition of the plant fillers. It is higher for samples containing WB and increases with the amount of filler in the samples. In the second stage of decomposition (with *T_max_*_2_ of approx. 388 °C), PBS degradation in composites occurs by hydrolysis with its simultaneous oxidation [11]. The last stage takes place at *T_max_*_3_ of approx. 440 °C and is associated with the oxidation of deposits produced in previous stages. *T*_50%_ is similar for samples with OP and WB.

### 3.3. Mechanical Properties

#### 3.3.1. Hardness

**Figure 10:**
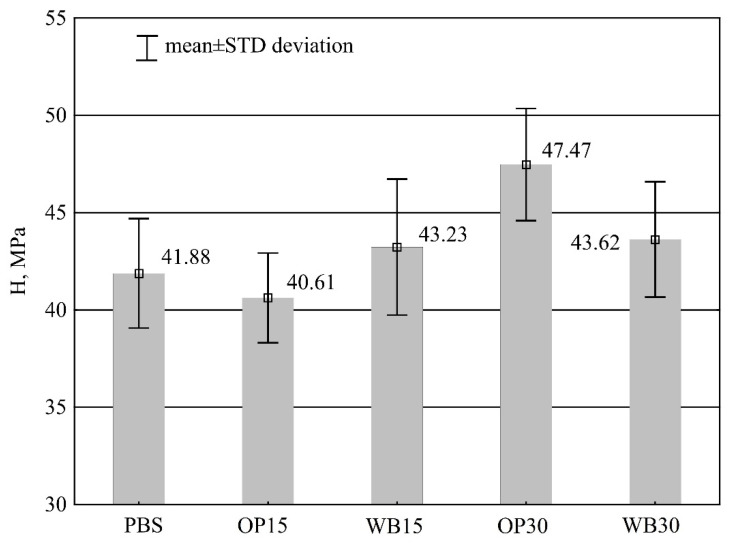
Hardness *H* samples of PBS and its composites with OP or WB.

Figure 10 presents the results of hardness tests of injection moldings obtained from the tested compositions. For both neat PBS and samples containing 15% wt. of the tested fillers and 30% wt. of WB, the obtained results are not statistically significantly different. The determined hardness remains in the range 38.2–48.7 MPa for the mentioned composition samples with an average of 42.5 MPa. The results of statistical analysis, however, showed a significant increase in hardness only for 30% wt. OP. The increase in hardness observed for OP was 5.59 MPa (13.3%) relative to neat PBS.

The influence of powder fillers on the hardness of composites depends on their distribution in the polymer matrix and the interaction at the -filler–matrix interface. Moreover, the mechanical properties as well as physical properties of fillers affect the composite hardness. The slight increase in hardness observed for compositions with 30% OP is typical of powdered fillers infused into a matrix with PBS [60,61].

There was no significant rise in the hardness of the other tested compositions with a higher filler content. This may be due to the lack of use of compatibilization between them and the PBS matrix. Only adequate compatibilization and uniformity of filler distribution in the polymer matrix ensures effective stress transfer and a consequent increase in hardness [62].

#### 3.3.2. Impact Strength

**Figure 11:**
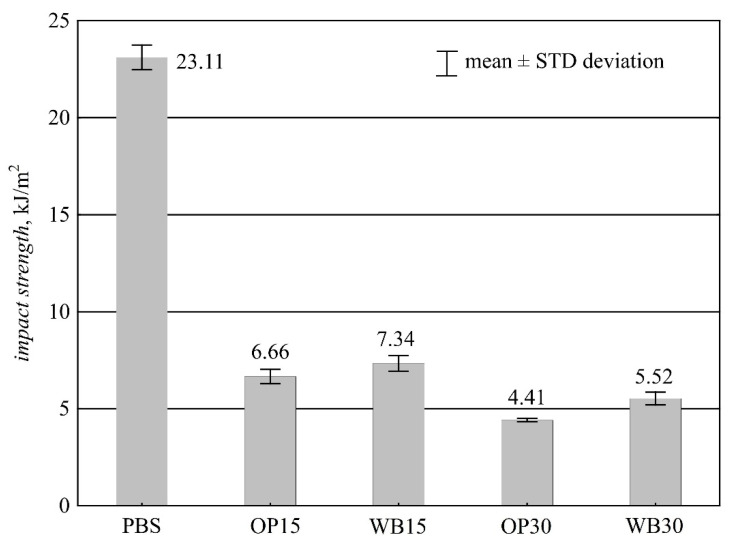
Notched impact strength of PBS and its composites with OP or WB.

Samples of neat PBS without notching did not crack at room temperature. Consequently, a decision was made to perform a measurement with a notch, which was also performed in the composition samples for comparability of results. The measurements carried out showed a sharp decrease in notched impact strength (Figure 11) as a result of the introduction of both WB and OP fillers into PBS. Even the lowest content of 15% wt. of both WB and OP resulted in a decrease in impact strength of about 70%. Tukey’s post-hoc statistical test showed no significant differences in notched impact strength between compositions containing 15% wt. WB and 15% wt. OP. For higher contents of 30% wt., the observed differences are statistically significant. Notched impact strength composition containing 30% wt. OP is 20.1% smaller than a composition containing the same amount of WB. On the other hand, when comparing this composition with neat PBS, we can observe a reduction by as much as 18.7 kJ/m^2^, i.e., by 80.9%. The observed fractures were brittle in nature. The crack resistance diminished considerably with filler content, because the energy of impact was mainly utilized for crack propagation [63,64]. This is because with a high filler content, the number of material defects that facilitate crack propagation increases. Moreover, the increased filler content led to an increased roughness of the fracture.

#### 3.3.3. Tensile Strength

**Figure 12:**
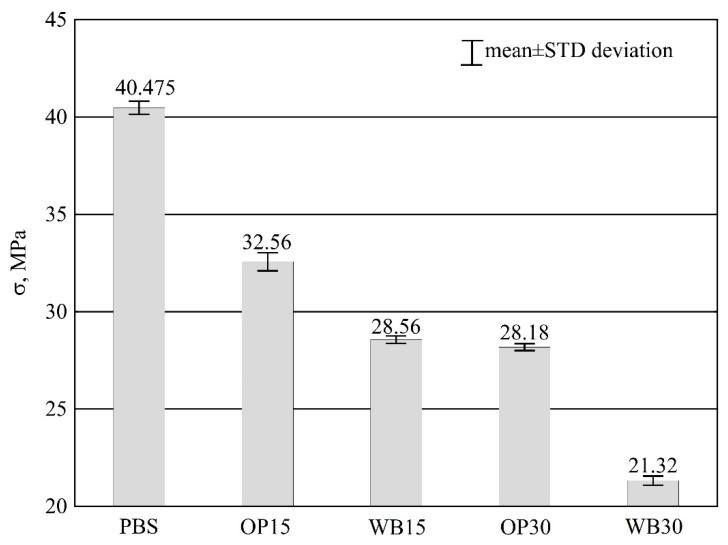
Tensile strength *σ* of PBS and its composites with OP or WB.

Increasing the content of the tested fillers in the compositions results in a noteworthy decrease in tensile strength *σ* (Figure 12), which for the samples with PBS is 40.48 ± 0.34 MPa.

The influence of WB content on the decrease in the strength of the compositions was clearly greater compared to OP. The addition of 15% wt. OP resulted in a 19.56% decrease in *σ*, while the introduction of 15% wt. WB resulted in tensile strength *σ* decreased by approx. 30%. However, the results of a post-hoc statistical analysis did not reveal any considerable differences in strengths of the composite samples containing 30% wt. OP and 15% wt. WB, which means that in the studied range it is possible to introduce twice the amount of OP into the composition compared to WB by maintaining the same tensile strength *σ*. For the WB30, containing the maximum content of WB, the greatest reduction in tensile strength of about 47% was recorded.

The tensile strength of polymer composites depends mainly on two factors: firstly, the quality of filler particle distribution in the polymer matrix, and secondly, the strength of interactions at the matrix–filler interface. Microcracks and discontinuities formation is caused by too small interaction forces. The low strength of the plant-based fillers used and the failure to use a compatibilizer lead to cavitation and debonding. Cavitation is the formation and expansion of empty spaces that constitute crack initiation sites [65]. Debonding occurs when filler particles separate from the matrix, which also leads to destruction. In the case of larger particles that are present in the fillers used, their debonding is easy and therefore the strength of the composition deteriorates as the filler content increases [66]. With an increase in the filler content, the amount of possible defects also increases, which can be a source of damage initiation in the material under tension. This is particularly important when it comes to the matrix made of PBS with moderate affinity to water and the hydrophilic fillers WB and OP [37,67]. A similar observation of decreasing tensile strength of the PBS-matrix biocomposites with increasing natural filler content was also reported by many other authors [13,18,19,23,24,37,67,68].

In addition, during processing, PBS, which is the matrix of the composition, is subject to processing shrinkage. This causes the plastic to shrink on the particles of the fillers used and leads to the tensile stresses in the matrix while filler particles are subjected to compressive stresses. As a result, the tensile strength of the composition is reduced since the stress concentration points to the formation of filler particles inside the PBS matrix [69,70].

#### 3.3.4. Young’s Modulus

**Figure 13:**
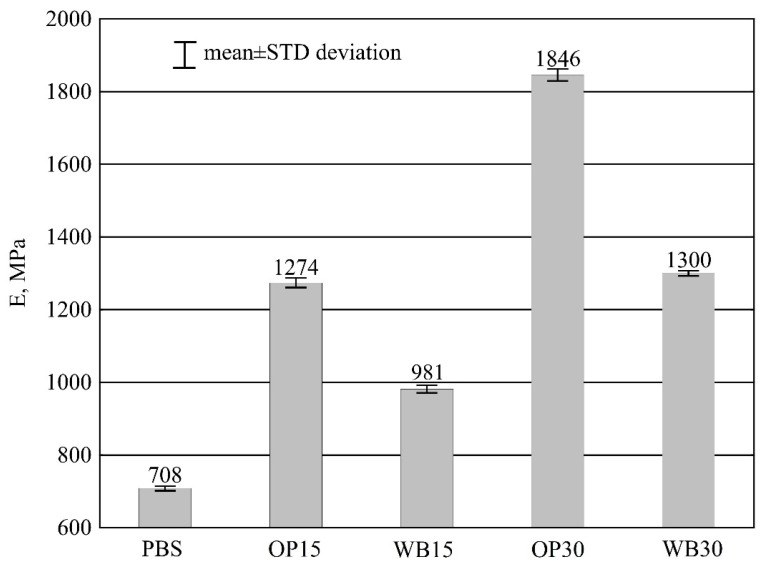
Young’s modulus E of PBS and its composites with OP or WB.

The introduction of the tested plant-based fillers in the form of small particles into PBS matrix leads to an increase in the stiffness of biocompositions. This is due to the restriction of the mobility of macromolecules through the dispersed phase. This behavior is typical of polymer composite materials with powdered mineral or natural fillers. [18,19,23,24,67,71]. Results of Young’s modulus E for the studied moldings are given in Figure 13. A statistical analysis of the results demonstrates considerable differences depending on the type of tested samples.

There is a noticeably greater increase in E value when OP are introduced into the composition compared to WB. Young’s modulus of the pure PBS was the lowest and averaged 708 ± 6 MPa. The use of 15% wt. WB resulted in a 38.56% increase in E value, while OP increased it by 79.94%. For the highest filler content of 30% wt., the increase in Young’s modulus E equaled 83.62% for WB and as much as 160.73% for OP, respectively. Roughly speaking, it can be said that the use of OP results in twice the increase in modulus E compared to WB. The observed increase in stiffness manifests itself in lower deformability, elasticity and plasticity, as well as increased brittleness, as confirmed by the results of the elongation measurements presented below.

#### 3.3.5. Elongation at Maximum Load

**Figure 14:**
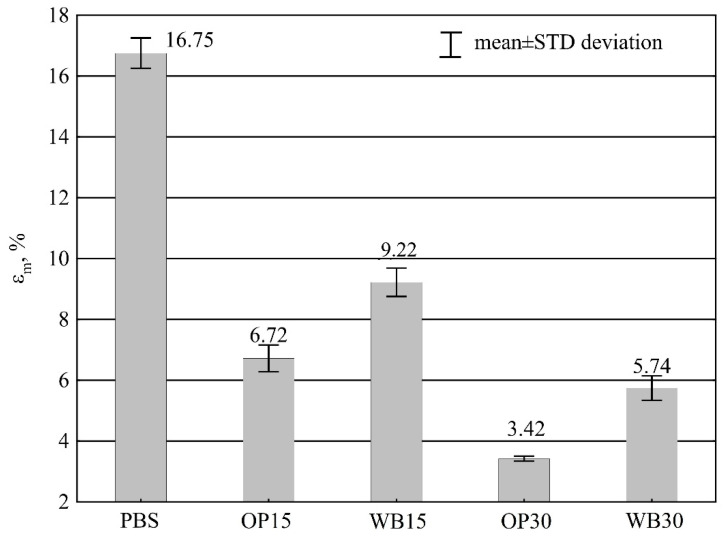
Elongation at maximum load *ε_m_* of PBS and its composites with OP and WB.

For all biocomposition samples tested, elongation at maximum load *ε_m_* corresponded to elongation at break because the samples broke in a brittle manner. In contrast, in the neat PBS specimens, once the maximum tensile stress was exceeded and a constriction was formed, further plastic deformation occurred until fracture occurred at about 100% strain. A statistical analysis showed significant differences between all types of the tested samples. The increase of the filler content causes a linear decrease in the elongation at maximum load *ε* (Figure 14). Significantly lower strain values are found in compositions containing OP compared to the same contents of WB. At the maximum mass content of fillers at 30%, the elongation value at maximum load *ε_m_* decreased by 11.01% for WB (which is 66% of the value for PBS) and by 13.33% for OP (which is 80% of the value for PBS). Neat PBS samples had a ductile fracture and significantly higher elongation at break of 16.75 ± 0.5%, with a characteristically very long gate.

This nature of changes in elongation at maximum load causes a significant reduction in deformability, which is a consequence of enhancement in the stiffness and brittleness of the biocomposition. The deterioration of the deformability of PBS compositions with the introduction of natural fillers such as pistachio shell, grape and apple pomace, bamboo and wood flour, is also described in other studies [19,23,24,67,71].

As in the case of impact strength, it can be observed that the fracture mode changed from ductile to brittle. Starting from 15% content of the tested plant fillers, the gate did not occur and brittle fractures with high roughness were obtained.

## 4. Conclusions

The conducted study on the biocompositions of PBS and OP or WB fillers showed that the content and type of filler influenced their properties under analysis.

Samples with the same mass filler content made with OP had a higher density compared to those obtained with WB. A slight increase in hardness was observed only for compositions with 30% OP, which is typical of powder fillers introduced into a polymer matrix. Notched impact strength measurements (the neat PBS samples without notching did not break) showed that even the lowest content of 15% wt. of both WB and OP fillers caused a decrease in impact strength by about 70%. At 30% wt., notched impact strength of the composition containing OP is 20.1% lower than the composition containing the same amount of WB.

The glass transition temperatures of compositions with 15% filler content are slightly lower than those with 30% filler content. The biocompositions degree of crystallinity is higher than for neat PBS and increases with OP or WB content increased. Compositions with WB are characterized by a higher degree of crystallinity than those with OP. The thermal resistance of compositions with WB and OP is lower than that of pure PBS and does not exceed 300 °C. The thermal resistance in oxidative conditions of compositions with OP is lower than the corresponding compositions with WB, which is due to the different thermal resistance of WB and OP.

Increasing the content of the tested fillers also causes a considerable decrease in the tensile strength of the compositions. The WB presence causes a markedly greater decrease in tensile strength compared to OP. It has been observed that in the studied range of filler content it is possible to introduce twice the amount of OP into the composition compared to WB by maintaining the same tensile strength.

There is also a noticeably greater increase in Young’s modulus E value when OP is introduced into the composition compared to WB. For the highest filler content of 30% wt., the increase in Young’s modulus equaled 83.62% for WB and as much as 160.73% for OP, respectively, compared to neat PBS. Roughly speaking, it can be said that the use of OP results in twice the increase in modulus E compared to WB.

Due to brittle fracture of all tested samples, elongation at maximum load *ε_m_* corresponded to elongation at break. A proportional decrease in the value of elongation at maximum load *ε_m_* was observed with the increasing filler content. Significantly lower strain values were found in compositions containing OP compared to the same contents of WB. Neat PBS samples, on the other hand, were characterized by a plastic breakthrough with a very long gate and much higher elongation at maximum load.

The biocomposites under study can be used for the production of everyday use objects, regardless of the observed deterioration in some properties. These can be reusable or single-use products, which after use will go to a composting plant, where they will biodegrade. The possibility of using high contents of both OP and WB fillers allows for a significant reduction in material costs in the industry. A promising direction for further research is the antimicrobial properties of OP compositions. The natural bioactive compounds contained in OP can most likely improve the antimicrobial and antibiofilm properties of products. The compositions may then prove to be desirable ingredients for creating environmentally friendly packaging materials.

## Figures and Tables

**Table 1:** Injection molding conditions in the manufacture of the biocomposite samples.

Quantity	Value
Temperature heating zones	I–125 °C, II–145 °C, III–155 °C, IV–160 °C
Temperature feed zone	30 °C
Temperature injection nozzle	155 °C
Temperature of the mold	25 °C
Maximum injection pressure	80 MPa
Polymer flow rate	20 cm^3^/s
Packing pressure	60–80 MPa
Packing time	15 s
Cooling time	20 s

**Table 2:** The research plan.

Composite Sample	PBS Contents, % wt.	OP Contents, % wt.	WB Contents, % wt.
PBS	100	0	0
OP15	85	15	0
WB15	85	0	15
OP30	70	30	0
WB305	70	0	30

**Table 3:** Melting point (*T_m_*), crystallization (*T_c_*), and glass transition (*T_g_*) temperatures, the enthalpy of melting (Δ*H_m_*), and the degree of crystallinity (*X_c_*) of PBS and composites with WB and OP, based on DSC thermograms.

Sample	Heating I				Cooling	Heating II			
	*T_g_* [°C]	*T_m_* [°C]	Δ*H_m_* [J/g]	*X_c_* [%]	*T_c_* [°C]	*T_g_* [°C]	*T_m_* [°C]	Δ*H_m_* [J/g]	*X_c_* [%]
PBS	−25.6	121.3	72.39	65.63	86.4	−31.7	118.5	70.03	63.49
OP15	−25.1	121.9	71.32	76.07	82.1	−33.2	116.7	59.05	62.98
WB15	−34.4	118.6	81.5	87.16	79.6	−33.6	116.7	69.05	73.65
OP30	−29.5	119.9	72.15	93.45	78.0	−30.2	119.2	51.89	67.21
WB30	−31.6	118.4	70.0	90.66	78.4	−29.5	118.0	61.19	79.25

**Table 4:** Parameters describing the thermal resistance of PBS, WB, OP and biocomposite materials, based on TG and DTG curves.

	*T*_5%_ [°C]	*T*_50%_ [°C]	*T_max_*_1_ [°C]	Δ *m*_1_ [%]	*T_max_*_2_ [°C]	Δ *m*_2_ [%]	*T_max_*_3_ [°C]	Δ *m*_3_ [%]	*R_m_* [%]
WB	107	303	296	68.60	-	-	459	26.69	4.71
OP	85	316	245	29.46	307	36.13	416	26.14	8.27
PBS	308	385	-	-	396	97.50	480	4.20	0
OP15	270	379	242	5.42	388	84.40	423	8.25	1.93
WB15	292	381	299	7.28	385	83.06	448	10.19	0
OP30	239	378	242	10.83	388	83.17	441	4.32	1.68
WB30	248	378	293	16.9	389	72.50	448	9.51	1.09

## Data Availability

The data presented in this study are available on request from the corresponding author.

## Supplementary Materials

The following supporting information can be downloaded at: https://www.mdpi.com/article/10.3390/ma16206799/s1, Figure S1: Microscopic images of OP captured for morphology calculations; Figure S2: Microscopic images of WB captured for morphology calculations; Table S1: Selected parameters characterizing the size and shape of particles of ground onion peels (OP) and wheat bran (WB).

## Nomenclature

PBS	Poly(butylene succinate)
WB	Wheat bran
OP	Onion peels
FTIR	Fourier transform infrared (spectroscopy)
DSC	Differential scanning calorimetry
*X_c_*	Degree of crystallinity
Δ*H_m_*	Melting enthalpy
*T_m_*	Melting point
*T_c_*	Crystallization temperature
*T_g_*	Glass transition temperature
TG	Thermogravimetry
DTG	Derivative thermogravimetry
*T*_5%_, *T*_50%_	Temperature of 5% and 50% of mass loss
*T_max_*	Temperature of the maximum rate of mass loss
Δ *m*	Mass loss corresponding to T_max_
*R_m_*	Residual mass
*LCF*	Lignocellulosic filler
*ρ*	Density
*H*	Hardness
*σ*	Tensile strength
*E*	Young’s modulus
*ε_m_*	Elongation at maximum load
