# Supplementary material for: Preparation and Spectroscopic, Thermal, and Mechanical Characterization of Biocomposites of Poly(butylene succinate) and Onion Peels or Durum Wheat Bran
Source: Materials (Basel). 2023 Oct 21;16(20):6799. doi: 10.3390/ma16206799 (PMC10607975; doi:10.3390/ma16206799)
Supplement: Supplementary file 1 [file materials-16-06799-s001.zip › materials-2651823-supplementary.pdf]

Supplementary Materials

Table S1. Selected parameters characterizing the size and shape of particles of ground onion peels (OP) and wheat bran (WB).

Sample	Particles Counted	Area Mean [μm²]	Aspect Ratio Mean	Circularity Mean
OP-A	73504	89.60	0.628	0.813
OP-B	72785	125.00	0.618	0.801
OP-C	81426	117.66	0.617	0.796
OP-D	83079	108.12	0.622	0.803
WB-A	9281	522.61	0.644	0.828
WB-B	14398	472.18	0.647	0.834
WB-C	12594	593.90	0.648	0.834
WB-D	17111	536.61	0.648	0.839

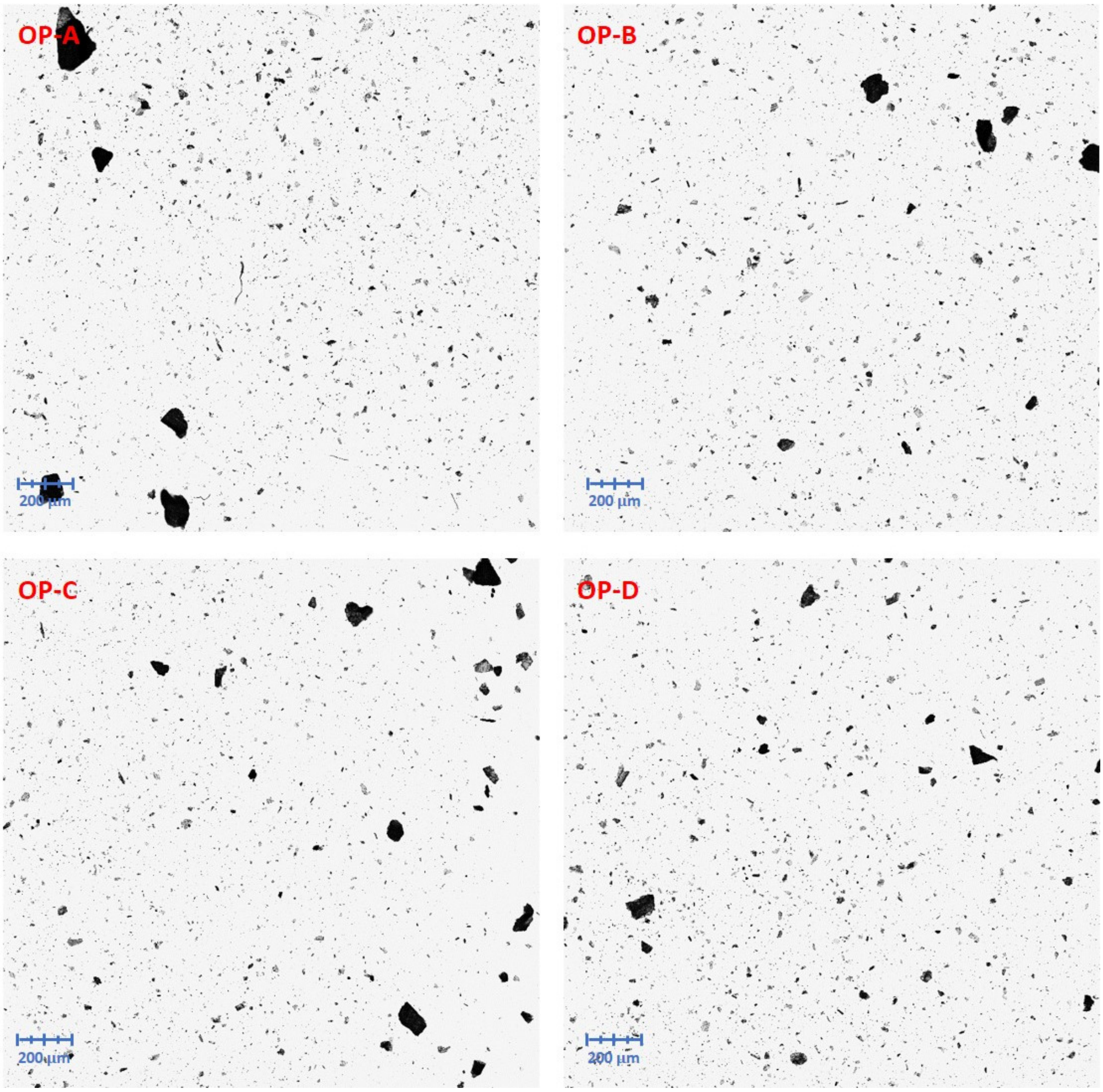

Figure S1. Microscopic images of OP captured for morphology calculations.

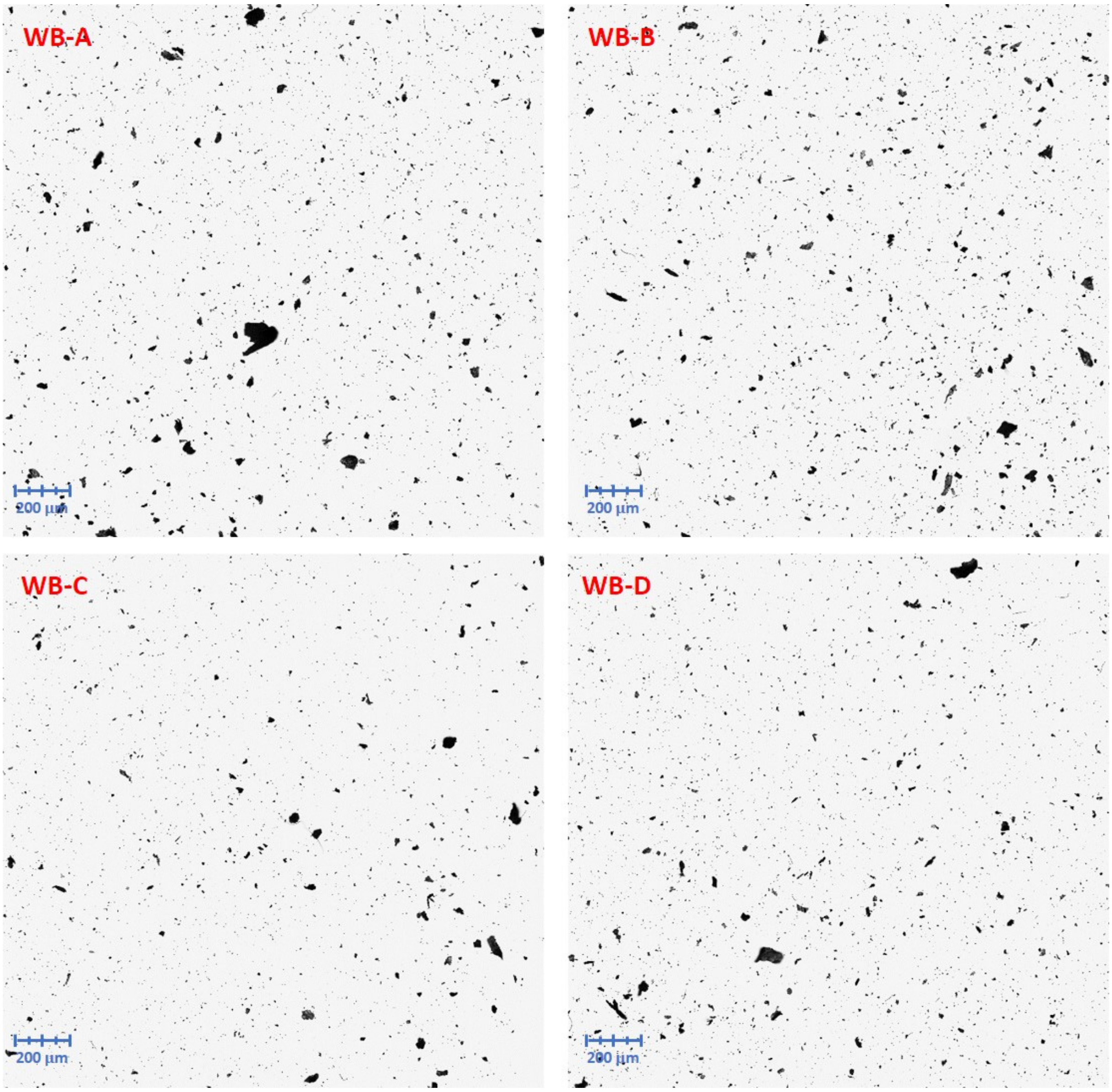

Figure S2. Microscopic images of WB captured for morphology calculations.
